# Branched-chain amino acid transferase type 2 (BCAT2) deficiency: Report of an eighth case and literature review

**DOI:** 10.1016/j.ymgmr.2025.101213

**Published:** 2025-04-09

**Authors:** Etienne Mondésert, Juliette Bouchereau, Manuel Schiff, Jean-François Benoist, Guilia Barcia, Boris Keren, Inès Mannes, Clément Pontoizeau, Charlotte Mansat, Apolline Imbard

**Affiliations:** a Department of Biochemistry, University Hospital of Montpellier, Montpellier, France; b Reference Center for Inborn Error of Metabolism, Department of Pediatrics, Necker-Enfants Malades Hospital, Assistance Publique-Hôpitaux de Paris, Paris, France; c Reference Center for Inborn Error of Metabolism, Department of Pediatrics, Necker-Enfants Malades Hospital, Assistance Publique-Hôpitaux de Paris and Université Paris Cité, Paris, France; d Inserm UMRS_1163, Institut Imagine, Paris, France; e Department of Biochemistry, Necker-Enfants Malades Hospital, Assistance Publique-Hôpitaux de Paris, Paris, France and Université Paris-Saclay, Paris, France; f Department of Genetics, Necker-Enfants Malades Hospital, Assistance Publique Hospital of Paris, Paris, France; g Department of Genetics, La Pitié-Salpêtrière Hospital, Assistance Publique Hospital of Paris, Paris, France and Sorbonne University, Paris, France; h Department of Pediatric Radiology, AP-HP, Bicêtre Hospital, Assistance Publique-Hôpitaux de Paris, Le Kremlin-Bicêtre, France; i Department of Pediatric Neurology, Bicêtre Hospital, Assistance Publique-Hôpitaux de Paris, Le Kremlin Bicêtre, France and Department of General Pediatrics, Paris-Saclay Hospital, GHNE, Orsay, France

**Keywords:** BCAT2, Branched-chain amino acid, Valine, Pyridoxine, Inborn error of metabolism

## Abstract

Branched-chain amino acid transferase type 2 (BCAT2) deficiency is a rare autosomal recessive genetic condition, with only seven cases described to date. It results in an elevation of branched-chain amino acid (BCAA) plasma concentrations, predominantly on valine, with normal concentration of plasma allo-isoleucine and urine branched-chain α-keto acids (BCKA). Despite this constant biochemical feature, clinical consequences remain unclear with heterogeneous and far less severe than maple syrup urine disease (MSUD) reported phenotypes, one individual being even asymptomatic.

We report herein the eighth case of genetically confirmed BCAT2 deficiency, accompanied by a literature review and a discussion about the potential pathogenicity of this condition.

An 11-year-old boy presented with a rapidly reversible initial acute neurological episode suggesting an epileptic seizure. Abnormalities on cerebral magnetic resonance imaging and suspicion of cognitive impairment led to further metabolic investigations. BCAT2 deficiency has been mentioned in front of increased BCAAs (valine = 1667 μmol/L, leucine = 701 μmol/L, isoleucine = 561 μmol/L). A homozygous novel nonsense variant on *BCAT2* (c.34C > T, p.Arg12*) was found on whole exome sequencing. After oral pyridoxine supplementation (200 mg/day), a decrease in BCAA concentrations was observed (valine = 984 μmol/L, leucine = 462 μmol/L, isoleucine = 302 μmol/L).

Laboratory and imaging findings were consistent with previously reported cases. However, clinical presentation of this case was atypical and could be related with epilepsy, although no other variant on epilepsy genes have been found. The relation between BCAT2 deficiency and these clinical findings is at this stage debated with regard to phenotypic variability. Further case-studies are needed to expand the knowledge about this condition.

## 1 Introduction

Branched-chain amino acid transferases (BCAT, EC 2.6.1.42) are enzymes that catalyze the reversible first step of branched-chain amino acids (BCAA) catabolism in which the BCAA are converted to their respective branched-chain α-keto acids (BCKA). There are two BCAT isozymes: the cytoplasmic BCAT1 and the mitochondrial BCAT2. BCAT2 is the most widely distributed of the two isoforms, especially in the brain [1]. *BCAT2* gene encodes a 392 amino acids (AA) protein, composed of a 27 AA mitochondrial targeting sequence and a 365 AA protein sequence [2]. The active form of the protein consists of a homodimer of two 365 AA monomers, each comprising a small (residues 1–170) and a large (residues 182–365) domain linked by an inter-domain loop (residues 171–181). Pyridoxal-5-phosphate (PLP) acts as a cofactor of BCAT2, covalently bound to Lys202 (Schiff base) [3]. Reactive cysteines 315 and 318 form the CXXC center of BCAT2, which acts as an essential regulation domain [2].

After BCAA transamination into BCKA, the branched-chain α-keto acid dehydrogenase complex (BCKDH), a multi-subunit structure of enzymes localized on the inner mitochondrial membrane, irreversibly decarboxylates BCKA. Defects in BCKDH activity lead to maple syrup urine disease (MSUD), a well-recognized metabolic disorder. Classical MSUD results in severe neonatal encephalopathy caused by the accumulation of BCAA (especially leucine) and BCKA in the tissues.

To our knowledge, only seven cases of genetically confirmed BCAT deficiency have been reported, all due to BCAT2 deficiency [[4], [5], [6], [7]]. The biochemical profile shows an isolated plasma elevation of BCAA, predominantly on valine in all patients. Clinical symptoms were less severe and acute than those observed in MSUD, ranging from neurological signs such as chronic headache, intellectual disability or autism spectrum disorder in childhood or early adulthood to a fully asymptomatic 37-year-old adult patient. The pathogenicity of BCAT2 deficiency seemed questionable as clinical presentation and treatment response were highly variable. Moreover, two asymptomatic newborns were diagnosed thought neonatal screening, highlighting the need to clarify the potential clinical features of this disorder [6,7].

In this article, we report a novel case of BCAT2 deficiency associated with a peculiar clinical presentation and a new variant and propose an updated review of the literature on this rare inborn error of metabolism.

## 2 Case presentation

An eleven-year-old boy born to consanguineous parents, was initially admitted to the emergency department for a stroke suspicion. He presented with right facial paralysis and dysarthria on awaking after a nap, in a context of lack of sleep. Shortly afterwards, the patient was found to be agitated with disrupted alertness and had an episode of fever (38.2 °C), which led to a lumbar puncture to rule out meningoencephalitis. A slightly increase in lactate was observed in cerebrospinal fluid (CSF) (2.13 mmol/L, normal ranges: 1.1–2.1 mmol/L) and venous blood (1.8 mmol/L normal range: 0.5–1.6 mmol/L). Plasma C-reactive protein level was below 5 mg/L, CSF viral and bacterial assays were negative as were blood autoantibody detection panels and urine toxicology screening. Symptoms resolved spontaneously within a few hours, and the patient was transferred to a neurology department.

**Fig. 1:**
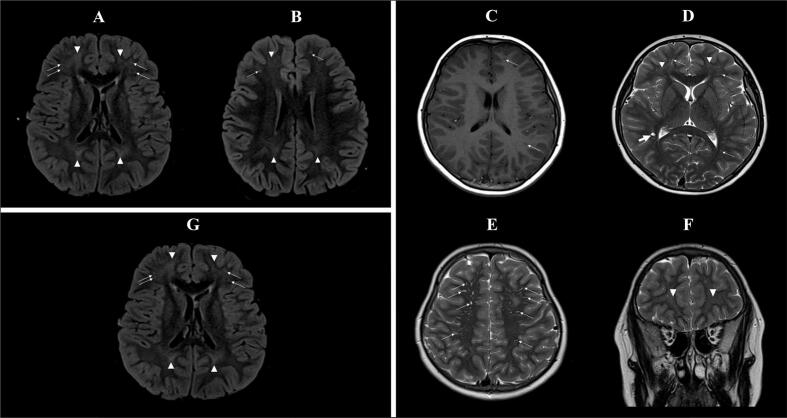
Magnetic Resonance Imaging (MRI) performed at admission (**A, B**) at day 5 (**C, D, E, F**) and 6 months after vitamin supplementation (**G**). **A.** Axial T2 FLAIR weighted imaging during the acute phase (at admission): symmetric mild increased FLAIR signals in bilateral fronto-parietal white matter (arrowheads), associated with punctiform high FLAIR signals in the frontal white matter, corresponding to perivascular spaces (arrows). **B.** Axial T2 FLAIR weighted imaging during the acute phase (at admission): symmetric mild increased FLAIR signal in bilateral fronto-parietal white matter (arrowhead), associated with punctiform high FLAIR signals in the frontal white matter (arrows), corresponding to perivascular spaces. **C.** Axial T1 weighted imaging 5 days after admission: no associated decreased T1 signal in bilateral fronto-parietal white matter lesions; visibility of some linear perivascular spaces (arrows). **D.** Axial T2 weighted imaging 5 days after admission: symmetric mild increased T2 signal in bilateral fronto-parietal white matter (arrowheads), associated with numerous linear (arrows) or cystic (bold arrow) perivascular spaces. **E.** Axial T2 weighted imaging 5 days after admission: numerous linear perivascular spaces (arrows). **F.** Coronal T2 weighted imaging 5 days after admission: symmetric mild increased T2 signal in bilateral anterior frontal white matter (arrowheads). **G.** Axial T2 FLAIR weighted 6 months after vitamin supplementation: stability of the symmetric mild increased FLAIR signal in bilateral fronto-parietal white matter (arrows), associated with punctiform high FLAIR signals in the frontal white matter (arrowheads), corresponding to known enlarged perivascular spaces.

A first MRI (Fig. 1A and B) was performed in emergency to rule out stroke: no recent ischemic lesion was found but there were some mild and nonspecific increased FLAIR signals in the frontal and parietal white matter. There were also some enlarged perivascular spaces (Virchow-Robin spaces) in the periventricular white matter. A second MRI (Fig. 1C,D, E, F) was performed at day 5 after admission and confirmed the stability of theses signal abnormalities with no abnormal metabolite at spectroscopy. An electroencephalogram (EEG) performed four days after the acute episode showed an asymmetry between left and right hemispheres (normal alpha wave and slower theta wave) but without clear abnormalities of an epileptic nature.

Intellectual disability was suspected during the hospitalization because of the patient's reading and arithmetic deficits for his age. However, the suspicion was nuanced because the patient had hardly ever attended school due to a complex social context. A neuropsychological assessment (WISC-V, TEACh) carried out after hospitalization revealed a heterogeneous profile, with borderline and very low indices (Wechsler scale index between 61 and 89) and attention difficulties.

**Fig. 2:**
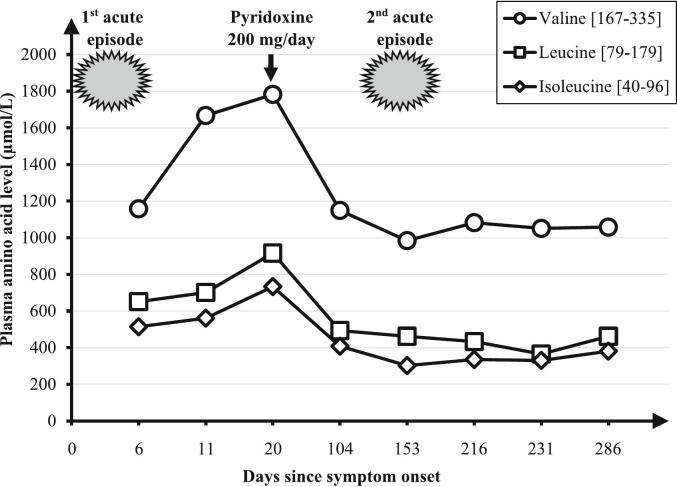
Valine, leucine and isoleucine plasma levels (μmol/L) evolution since symptom onset. Normal ranges (μmol/L) of the three analytes are indicated. The two acute episodes and introduction of oral pyridoxine (vitamin B6) supplementation (200 mg/day) are also represented.

The metabolic workup sampled during the acute episode showed an isolated increase in plasma BCAA (Table 1, Fig. 2) (valine = 1158 μmol/L [normal ranges: 167–335], leucine = 651 μmol/L [normal ranges: 79–179], isoleucine = 514 μmol/L [normal ranges: 40–96]), without an allo-isoleucine increase (2 μmol/L). Urine amino acid and organic acid profiles were normal (no elevated BCKA). The plasma amino acid profile was confirmed in two further samples taken 10 days apart, with higher plasma valine concentrations of 1667 and 1782 μmol/L. BCAA levels measured on the first chromatography were potentially underestimated due to concomitant carbohydrate perfusion with protein restriction. Whole genome sequencing (WGS) revealed a previously undescribed homozygous nonsense variant on *BCAT2* (c.34C > T, p.Arg12*). The variant was classified as likely pathogenic using American College of Medical Genetics and Genomics (ACMG) [8] classification meeting the PVS1 and PM2 (GnomAD frequency = 0.000034) criteria. Parents were both heterozygous carriers of this *BCAT2* variant. No additional pathogenic or likely pathogenic variants according to the ACMG criteria were found on WGS.

**Table 1:** Plasma amino acid values at admission.

Amino acid	Value	Unit	Reference ranges
Alanine	184	μmol/L	152–472
Arginine	104	μmol/L	35–115
Asparagine	47	μmol/L	36–60
Citrulline	49	μmol/L	18–42
half-Cystine	104	μmol/L	53–113
Glutamine	565	μmol/L	331–715
Glutamic acid	29	μmol/L	29–139
Glycine	181	μmol/L	134–290
Histidine	71	μmol/L	55–107
Isoleucine	514	μmol/L	40–96
Leucine	651	μmol/L	79–179
Lysine	134	μmol/L	118–187
Methionine	34	μmol/L	17–37
Ornithine	75	μmol/L	17–115
Phenylalanine	70	μmol/L	26–86
Proline	206	μmol/L	42–322
Serine	105	μmol/L	77–158
Taurine	55	μmol/L	31–155
Threonine	135	μmol/L	42–226
Tryptophan	75	μmol/L	25–80
Tyrosine	63	μmol/L	36–104
Valine	1158	μmol/L	167–335
α-Aminoadipic acid	1	μmol/L	<5
Argininosuccinic acid	0	μmol/L	<1
Allo-Isoleucine	2	μmol/L	<5
Aspartylglucosamine	0	μmol/L	<1
δ-Aminolevulinic acid	0	μmol/L	<1
Homocitrulline	0	μmol/L	<5
Homocystine	0,1	μmol/L	<0,5
Hydroxyproline	49	μmol/L	<45
Pipecolic acid	1,3	μmol/L	<5
Sulfocysteine	2	μmol/L	<5

The potential benefits of a low-protein diet was considered and discussed with the family but deemed unfeasible due to the social context. Oral pyridoxine (vitamin B6, pyridoxal phosphate being pyridoxine active form and the cofactor of BCAT2) supplementation (200 mg/day) was started two weeks after hospitalization.

Three months after the initial hospitalization, the patient was admitted for a new episode of facial paralysis with unilateral lower limb paresthesia and dysarthria again on awaking after a nap. He was quickly discharged from the emergency department as the symptoms were completely reversible. A new EEG showed bi-fronto-temporal spike-and-waves during sleep, suggestive of incipient epilepsy. No antiepileptic treatment was started, given the rarity of the episodes.

*Re*-evaluation was performed 3 months later with EEG, which showed the same abnormalities, and at 6 months with an unchanged brain MRI (Fig. 1G) and the same white matter signal abnormalities. A new neuropsychological assessment (TEACh) showed that visual attention performance was still affected, but had improved compared with the previous assessment, as had processing speed.

Throughout the follow-up, BCAA plasma concentrations initially decreased after 3 months of oral pyridoxine supplementation (valine: −35.5 %, leucine: −46.2 %, isoleucine: −44.3 %), but remained stable and still above normal ranges thereafter (Fig. 2). Additionally, no other acute clinical episode was reported.

## 3 Literature review and discussion

**Fig. 3:**
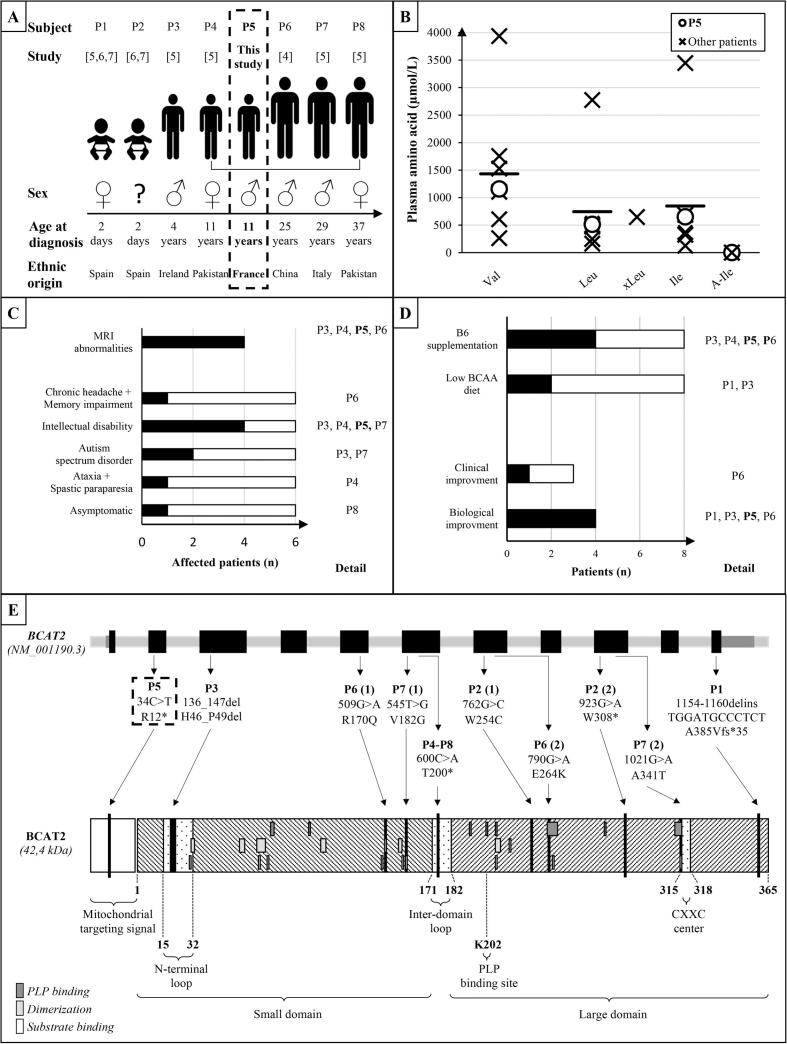
Characteristics of the present case (P5) and the seven published cases (P1-P4, P6-P8) of BCAT2 deficiency. **A.** Epidemiological findings. P4 is the daughter of P8. **B.** Plasmatic BCAA levels at diagnosis (Val: valine, Leu: Leucine, xLeunnnnnnnn: Leucine + Isoleucine, Ile: Isoleucine), with mean indicated by a black bar. Levels of our patient are highlighted by circles. **C.** Clinical and MRI findings of the 6 adult patients. **D.** Treatments and evolution. For figs. C and D, black bars indicate number of affected or treated patients. **E.** Summary of genetic variants associated with *BCAT2* deficiency and their impact on BCAT2 protein structure. Schematic *BCAT2* gene representation shows exons (black), introns (light gray), and non-coding exons (gray). For each patient mutations on nucleotide and protein sequences are shown. Schematic BCAT2 protein structure is represented with amino acid numeration starting after the 27 mitochondrial targeting sequence (white). Amino acids involved in PLP binding pocket (dark gray rectangles), amino acids involved in BCAT2 dimerization (light gray rectangles) and amino acids involved in substrate binding (white rectangles) are represented. Other important structural domains are shown in white with black dots. Amino acids impacted by nonsense (long black bars) and missense (small black bars) mutations are also represented.

Herein, we report the eighth case of genetically confirmed BCAT2 deficiency (Fig. 3A). Wang et al. reported the first case in 2015 [4], followed by a case-series of 5 patients published in 2019 [5], and a last case found through neonatal screening [6,7]. As in all other cases, isolated predominant hypervalinemia with milder elevations of leucine and isoleucine was the main laboratory finding (Fig. 3B). At the time of diagnosis, minimum-maximum levels of valine, leucine and isoleucine were equal to 263–3935 μmol/L, 163–2774 μmol/L and 126–3446 μmol/L respectively. Situations with elevated BCAA such as prolonged fasting, and diabetes mellitus were quickly ruled out given the massive, persistent and isolated increase in BCAA. MSUD and E3 deficiency were also ruled out by the absence of plasma allo-isoleucine and BCKA elevations in urine, the absence of prolonged neurological impairment, and the absence of pathogenic variants found in *BCKDHA, BCKDHB, DBT, PPM1K or DLD* genes.

Despite its constant biological abnormalities, BCAT2 deficiency has been associated with a broad phenotypic spectrum and variable ethnic origins (Fig. 3C), with manifestations in childhood and early adulthood. The first described case was a 25-year-old man who presented with a chronic headache and memory impairment history [4], while three cases were children with intellectual disability [5]. Two of these patients exhibited moderate symptoms; the two others had a severe chronic presentation with other associated symptoms (autism spectrum disorder with severe language disability, microcephaly with ataxia, and spastic paraplegia). Additionally, one 37-year-old adult patient diagnosed through family genetic investigation was asymptomatic, as well as two newborns diagnosed after neonatal screening [[5], [6], [7]].

Our patient presented two acute and reversible neurological episodes with EEG abnormalities which could be part of early focal epilepsy of undetermined origin. As for three other patients, intellectual disability was the clinical presentation leading to metabolic work-up and BCAT2 deficiency diagnosis. The intellectual disability is however very questionable for our patient due to episodic schooling. Brain magnetic resonance imaging (MRI) data are sparse (Fig. 3C) but three patients presented white matter abnormalities. One patient had large and well demarcated T2/FLAIR hyperintensities in the periventricular white matter; the others also had nonspecific T2/FLAIR hyperintensities in the white matter, less extensive. The white matter of our patient was also pathological, showing mild increased T2/FLAIR signal especially in the frontal and parietal lobes, associated with some enlarged perivascular spaces (Fig. 1).

Two treatment strategies were tested (Fig. 3D): pyridoxine (vitamin B6) supplementation (three patients) since PLP acts as a cofactor of BCAT2 [3] and BCAA-restricted diet (one patient), or both (one patient). Although a significant decrease in plasma BCAA was reported in four cases (mean ± standard deviation valine level before vs after treatment: 2019 ± 1390 vs 527.4 ± 379.3 μmol/L), clinical improvement was noted in only one patient treated by pyridoxine supplementation (200 mg/day), two patients remained symptomatic, and one patient diagnosed on neonatal screening remained asymptomatic at 2.5 years of age.

Genetic analysis showed private familial *BCAT2* variants, with a history of consanguinity in 2 cases including ours (Fig. 3E). As shown in Fig. 2E, all variants had an important putative in silico repercussion on BCAT2 monomer structure or activity. The p.Thr200* variant removes the entire large domain comprising the PLP binding site and CXXC center. p.Ala385Valfs*35 is responsible for the loss of a stabilizer beta-sheet formed by the seven last residues. Western-blot analysis of protein extracted from cellular and nuclear lysates of fibroblasts from this patient showed absence of BCAT2 protein [5]. p.His46_Pro49del alters the N-terminal loop (residues 15–32) which is also a critical structural domain [2]. BCAT2 activity (valine decarboxylation rate) measured was found reduced in fibroblasts from the patient carrying p.His46_Pro49del variant. Apart from W254C, which creates a potentially unstable reactive cysteine, all other mutations affect dimerization sites or the PLP/substrate binding pocket. The p.Arg12* variant found in our patient can produce a mislocated protein or no protein at all. We did observe a partial effectiveness of pyridoxine supplementation on BCAA plasma level reduction suggesting the production of a functional mislocated protein with an alternative translation initiation start site. Three variants (p.Arg170Glu, p.Gln264Lys and p.His46_Pro49del) have been functionally studied using recombinant purified protein or cultured skin fibroblasts showing a decreased BCAT2 activity (valine carboxylation rate) for the 3 variants [4,5].

Given the low specificity of the symptoms, the presence of a concomitant genetic disease must be excluded. One patient had a nonsense mutation in the *B4GALNT1* gene that could partially explain his phenotype. Of the seven previously described BCAT2 deficient patients, only two underwent a targeted whole exome sequencing [5]. In our own case, we performed WGS that did not show any other mutation in known intellectual disability and congenital epilepsy related genes.

Three cases of so-called idiopathic hypervalinemia without an increase of leucine and isoleucine were also reported but weren't genetically investigated [9,10]. Symptoms included failure to thrive, vomiting, nystagmus, hyperkinesia, physical and intellectual disability, and muscular weakness and were responsive to low-valine diet in one case [11]. As no enzymatic deficiency or gene has been associated to these cases, BCAT2's involvement uncertain in these cases.

Overall, it is unclear whether BCAT2 deficiency biochemical abnormalities (accumulation of BCAA and especially valine) are responsible for the clinical features described in BCAT2 deficiency. As mentioned above, reported symptoms are very heterogeneous, with one adult being asymptomatic (Fig. 3C). A variability in BCAA concentrations is also encountered amongst BCAT2 deficient patients (Fig. 3B) with baseline valine ranging from 263 to 3935 μmol/L. Moreover, leucine concentrations of some BCAT2 deficient patients are comparable to those of MSUD patients, in a range associated with altered cognitive function (decreased intellectual quotient) [12]. One of the patients had a plasma leucine concentration of 3446 μmol/L, comparable or even higher than concentrations observed in MSUD neonatal cases. However, neurotoxicity in MSUD is probably mediated by not only leucine but also and chiefly by BCKA accumulation [13]. Their absence in BCAT2-deficient patients could possibly explain the different clinical phenotypes. Furthermore, the cytosolic isoform BCAT1 might ensure residual BCAA metabolism in case of BCAT2 deficiency such that leucine accumulation could be then less severe at the cellular level, even if BCAT1 is less widely distributed across organs than BCAT2 [1]. That could explain the discrepancies between increased leucine plasma concentrations and the absence of severe acute neurological impairment in BCAT2 deficient patients. Regarding potential valine toxicity, an interesting case-report described a dietetic supplementation error resulting in a plasma valine level of 8380 μmol/L and an overall clinical worsening (reduced responsiveness, lip-smacking movements, abnormal muscle tone) but again in a 9-month-old patient with MSUD [14].

The presence of brain MRI lesions in four patients, including ours, could be an argument for slow and chronic toxicity due to valine or BCAA accumulation. The white matter signal changes observed in all four patients remain nonspecific: they do not appear progressive and do not present any specific lesion pattern [15]. Notably, perivascular spaces were enlarged in the periventricular white matter, but this can also be seen in healthy individuals [16]. These MRI abnormalities also differ from decompensated MSUD MRI abnormalities that are mostly located in the corticospinal tract, cerebellar peduncles and thalami, with spectroscopy abnormal signals [17].

A mice model of BCAT2 deficiency was obtained using N-ethyl-N-nitrosourea (ENU)-induced mutagenesis [18], with a homozygous splicing site mutation leading to exon 2 skipping. Affected mice presented BCAA increase, predominant hypervalinemia, and clinical signs (failure to thrive, weakness with decreased spontaneous movements). Recently a more specific CRISPR-Cas9 BCAT2 knocked-out mice model was designed and showed increased mortality rate and a surprising BCKA accumulation compared to wild types or only BCAT1 knocked-out mice [19]. However, these models present important limitations: ENU-induced mutagenesis had potentially led to other mutations and arginine and BCKA were increased. BCAT2 activity is completely abolished in CRISPR-Cas9 BCAT2 knocked-out mice, while a residual activity can be present in the case of human BCAT2 mutations.

In the event that BCAT2 is only a biochemical trait with no or minimal clinical impact, a perspective could be to study its inhibition as a substrate reduction therapy for treating MSUD. This was already studied for other inherited disorders of valine and isoleucine metabolism localized further down in the metabolic pathway with encouraging results [20].

Another crucial point to consider is the potential rise of cases identified through newborn screening, as programs constantly expand [21]. The value of treating these newborns with low-BCAA diet or pyridoxine supplementation remains unknown in the current state of the knowledge.

To conclude, BCAT2 deficiency is an autosomal recessive inherited condition characterized by a typical plasma amino acid profile with predominant hypervalinemia and without allo-isoleucine or BCKA elevation. Outside nonspecific cerebral MRI abnormalities, there is no clear argument for a link between BCTA2 deficiency and the observed symptoms in the eight described cases. Further studies and case descriptions with sufficient hindsight are needed to answer this question.

## CRediT authorship contribution statement

**Etienne Mondésert:** Writing – original draft, Data curation, Conceptualization. **Juliette Bouchereau:** Writing – review & editing, Resources, Investigation. **Manuel Schiff:** Writing – review & editing, Resources, Investigation. **Jean-François Benoist:** Writing – review & editing, Resources. **Guilia Barcia:** Investigation. **Boris Keren:** Investigation. **Inès Mannes:** Writing – review & editing, Resources, Investigation. **Clément Pontoizeau:** Writing – review & editing, Resources. **Charlotte Mansat:** Writing – review & editing, Resources, Investigation. **Apolline Imbard:** Writing – review & editing, Supervision, Conceptualization.

## Funding

This research did not receive any specific grant from funding agencies in the public, commercial, or not-for-profit sectors.

## Declaration of competing interest

The authors declare that they have no known competing financial interests or personal relationships that could have appeared to influence the work reported in this paper.

## Data Availability

Data will be made available on request.
